# Age-stratified anti-tuberculosis drug resistance profiles in South Korea: a multicenter retrospective study

**DOI:** 10.1186/s12879-020-05157-6

**Published:** 2020-06-23

**Authors:** Eung Gu Lee, Jinsoo Min, Ji Young Kang, Sung Kyoung Kim, Jin Woo Kim, Yong Hyun Kim, Hyoung Kyu Yoon, Sang Haak Lee, Hyung Woo Kim, Ju Sang Kim

**Affiliations:** 1 Division of Pulmonary, Allergy and Critical Care Medicine, Department of Internal Medicine, Bucheon St. Mary’s Hospital, College of Medicine, The Catholic University of Korea, Seoul, Republic of Korea; 2 Division of Pulmonary and Critical Care Medicine, Department of Internal Medicine, Daejeon St. Mary’s Hospital, College of Medicine, The Catholic University of Korea, Seoul, Republic of Korea; 3 Division of Pulmonary, Allergy and Critical Care Medicine, Department of Internal Medicine, Seoul St. Mary’s Hospital, College of Medicine, The Catholic University of Korea, Seoul, Republic of Korea; 4 Division of Pulmonology, Department of Internal Medicine, St. Vincent’s Hospital, College of Medicine, The Catholic University of Korea, Seoul, Republic of Korea; 5 Division of Pulmonary and Critical Care Medicine, Department of Internal Medicine, Uijeongbu St. Mary’s Hospital, College of Medicine, The Catholic University of Korea, Seoul, Republic of Korea; 6 Division of Pulmonary and Critical Care Medicine, Department of Internal Medicine, Yeouido St. Mary’s Hospital, College of Medicine, The Catholic University of Korea, Seoul, Republic of Korea; 7 Division of Pulmonary, Critical Care and Sleep Medicine, Department of Internal Medicine, Eunpyeong St. Mary’s Hospital, College of Medicine, The Catholic University of Korea, Seoul, Republic of Korea; 8 Division of Pulmonary and Critical Care Medicine, Department of Internal Medicine, Incheon St. Mary’s Hospital, College of Medicine, The Catholic University of Korea, 56, Dongsu-ro, Bupyeong-gu, Incheon, 21431 Republic of Korea

**Keywords:** Drug-resistant tuberculosis, Isoniazid, Rifampicin, Fluoroquinolone, Multidrug-resistant tuberculosis, Elderly population

## Abstract

**Background:**

The emergence of drug-resistant tuberculosis (DR-TB) is a major healthcare concern worldwide. Here, we analyzed age-related trends in DR-TB rates in South Korea.

**Methods:**

Drug susceptibility test results were collected from patients with culture-confirmed TB between 2015 and 2018 from eight university-affiliated hospitals. Patients were divided into three subgroups: younger (15–34 years), middle (35–59 years), and older (≥60 years) to compare drug-resistance patterns. To evaluate trends in age-stratified drug-resistance, chi-square test for linear trends was performed.

**Results:**

Among enrolled native patients aged ≥15 years, 4.1% (179/4417), 1.2% (53/4417) and 7.2% (316/4417) were multidrug-resistant TB (MDR-TB), rifampicin-mono-resistant TB (RR-TB), and isoniazid-mono-resistant TB (Hr-TB), respectively. Proportions of Hr-TB cases were 5.4% (40/734), 7.2% (114/1593), and 7.8% (162/2090) in the younger, middle and older age groups, respectively. MDR/RR-TB case rates decreased significantly with age from 8.6% (63/734) in younger age group to 3.3% (68/2090) in older age group. Fluoroquinolone resistance was highest among second-line drugs, and there were no differences in resistance to fluoroquinolones and second-line injectable drugs among the three age groups.

**Conclusions:**

The number of MDR/RR-TB cases was highest in young patients. Effective public health interventions should include increased focus on rifampicin resistance in young patients.

## Background

Drug-resistant tuberculosis (DR-TB) is a major global public health concern [1]. A 95% reduction in TB mortality and 90% reduction in its incidence compared to that in 2015 should be achieved by 2035 according to the World Health Organization (WHO)‘s End TB strategy [2]. Preventing the spread of DR-TB is important for the elimination of TB [3]. Multidrug-resistant TB (MDR-TB), which is resistant to isoniazid (INH) and rifampicin (RIF), is another obstacle because of its high treatment costs and unsatisfactory outcomes.

Although anti-TB drug resistance rates declined after improved treatment efficiency in South Korea in the 1980s, nationwide drug surveillance conducted between 1994 and 2004 revealed that drug resistance had increased among new TB cases [4]. However, the nationwide trend of anti-TB drug resistance could not be estimated as this survey was discontinued and replaced with a new TB notification system [5]. The limited availability of data concerning drug resistance profiles thus hampers effective treatment of DR-TB.

The Korean War, which took place between 1950 and 1953, is regarded as the strongest causative factor for the TB epidemic in South Korea [6]. The period between 1965 and 1995 saw phenomenal economic growth and the expansion of national health insurance, leading to a dramatic decrease in TB incidence. After 2000, incidence stagnated for a decade despite continuous national efforts to control the disease [7]. Considering South Korea’s unique history of improved socioeconomic status and decreased TB incidence observed over a half century, we hypothesized that age and generation might represent multifactorial causes of emergence of drug-resistance, such as inappropriate regimens, use of lower-than-recommended doses, poor drug quality and poor treatment adherence [8], and the degree and status of TB exposure in the younger generation differ from that in the previous generation, which may consequently affect drug resistance profiles. We, therefore, conducted a multicenter cross-sectional study to analyze drug resistance patterns associated with age among culture-confirmed TB cases in South Korea.

## Methods

### Study design and data collection

We included native patients age ≥ 15 years who were diagnosed with culture-confirmed TB and who had available results of phenotypic drug-susceptibility test (DST) at eight university-affiliated hospitals in the Seoul metropolitan area and Daejeon between January 2015 and December 2018. Patients under the age of 15 and foreign-born patients were excluded. We excluded culture-confirmed cases without DST results, culture-negative cases, and clinically (radiologically, histologically or through a therapeutic response to anti-TB therapy) diagnosed cases. We retrospectively reviewed medical records and collected age, gender, history of previous TB treatment, location of TB, and phenotypic DST data. Previous TB history was assessed by physicians via patient history-taking. All the enrolled patients were received anti-TB treatment regimen based on the Korean TB guideline, which was first published in 2011. TB patients were initially treated with first-line regimen and later their treatment regimens were modified according to the DST results or clinical response.

### Culture-based phenotypic drug susceptibility test

All the AFB culture tests were performed prior to initiation of anti-TB treatment, and the DSTs were performed using the earliest *Mycobacterium tuberculosis complex* isolate. If AFB culture result after 3 months of anti-TB treatment was positive, the second DST was performed based on the Korean TB guidelines. If a patient had more than one DST result, only the earliest result was obtained. If a patient had DST results for both pulmonary and extra-pulmonary specimens, results for the pulmonary specimen only were used. DST was performed in a supranational reference laboratory (Korean institute of Tuberculosis, Osong, South Korea) or other commercial reference laboratories. Workflows used and references of critical concentrations for resistance were the same in all reference laboratories. The drug susceptibility of *Mycobacterium tuberculosis* isolates was determined using an absolute concentration method with Lowenstein-Jensen medium, as recommended by the WHO [9]. Anti-TB drugs used and their critical concentrations for resistance were as follows: INH, 0.2 μg/mL; RIF, 40 μg/mL; ethambutol, 2.0 μg/mL; rifabutin, 20 μg/mL; streptomycin, 10 μg/mL; amikacin, 40 μg/mL; kanamycin, 40 μg/mL; capreomycin, 40 μg/mL; ofloxacin, 2.0 μg/mL; levofloxacin, 2.0 μg/mL; moxifloxacin, 2.0 μg/mL; prothionamide, 40 μg/mL; cycloserine, 30 μg/mL; para-aminosalicylic acid, 1.0 μg/mL. Pyrazinamide susceptibility was determined via pyrazinamidase test.

### Definitions of variables

DR-TB cases were classified according to culture-based phenotypic DST results. DR-TB was defined as resistant to any anti-TB drug described, MDR-TB as resistant to both INH and RIF. RIF-mono-resistant TB (RR-TB) was defined as RIF-resistant but INH-susceptible, and INH-mono-resistant TB (Hr-TB) was defined as INH-resistant but RIF-susceptible. First-line drugs include INH, RIF, pyrazinamide, ethambutol, and streptomycin according to Korean TB guidelines [10]. New patients were defined as those never treated for TB or who had been prescribed anti-TB drugs for < 1 month, according to the WHO’s definition [11]. A patient with both pulmonary and extrapulmonary TB was classified as having pulmonary TB. We divided patients into three age groups based on socioeconomic background and TB status as follows: younger generation, aged 15 to 34 years; middle generation, aged 35 to 59 years, and older generation, aged 60 years or older (Table 1).

**Table 1:** Rationale of age stratification based on socioeconomic background and tuberculosis status of each birth year in South Korea

Age Group (Years)	Calendar year of birth	Socioeconomic background of each calendar year	TB status of each calendar year	National guideline for anti-TB treatment
15–34	After 1980s	Sustained economic growth with expansion of national health insurance	Sustained decrement of TB prevalence	Shorter regimen with additional use of rifampicin for 6–9 months
35–59	Between 1960s and 1970s	Spurt of economic growth	Marked by rapidly declining TB prevalence	Triple therapy using isoniazid and streptomycin for 18 months
≥60	Before 1950s	Before and after the Korean War	Probable explosion of TB epidemic in the Korean peninsular	Before implementing national TB control program

*Abbreviations*: *TB* tuberculosis

### Statistical analysis

Data are presented as numbers with percentages for categorical variables. To compare the differences between new and retreatment cases, we performed univariate analyses using binary logistic regression. Subsequently, we selected variables with p-values < 0.20 based on the univariate analysis and further performed multivariate binary logistic regression. To evaluate trends in age-stratified drug-resistance, extended Mantel-Haenszel chi-square test for linear trends were performed [12]. A *p*-value of 0.05 was considered statistically significant. All statistical analyses were performed using SPSS Statistics for Windows software (Statistical product and Service Solutions, ver. 20.0; IBM Co., Almonk, NY, USA).

### Ethics statement

This study was conducted in accordance with the Declaration of Helsinki. The Institutional Review Board of the Catholic Medical Center, the Catholic University of Korea approved the study protocol (XC19REDE0040) and waived the need for informed consent because no patients were at risk.

## Results

After excluding 18 patients aged < 15 years and 227 foreign-born patients, 4417 native patients with TB, aged ≥15 years, were included in this analysis. Of those, 15.8% (697/4417) had a prior history of anti-TB treatment and 15.5% (684/4417) had DR-TB (Table 2). Compared to the younger age group, adjusted odds ratios (ORs) for prior anti-TB treatment were 1.85 (95% Confidence Interval (CI) = 1.39–2.47) in the middle age group and 1.94 (95% CI = 1.47–2.56) in the older age group. Male gender (aOR = 1.75, 95% CI = 1.46–2.09) and DR-TB (aOR = 2.13, 95% CI = 1.74–2.59) were also significantly associated with prior anti-TB treatment.

**Table 2:** Demographic and clinical characteristics of enrolled patients and their association with history of anti-tuberculosis treatment

	Retreatment (*n* = 697)	New (*n* = 3720)	All (*n* = 4417)	OR (95% CI)	*p-value*	aOR (95% CI)	*p-value*
Age (years)							
15–34	68 (9.8%)	666 (17.9%)	734 (16.6%)				
35–59	275 (39.5%)	1318 (35.4%)	1593 (36.1%)	2.04 (1.54–2.71)	0.000	1.85 (1.39–2.47)	0.000
≥ 60	354 (50.85)	1736 (46.7%)	2090 (47.3%)	2.00 (1.52–2.63)	0.000	1.94 (1.47–2.56)	0.000
Male gender	495 (71.0%)	2130 (57.3%)	2625 (59.4%)	1.83 (1.53–2.18)	0.000	1.75 (1.46–2.09)	0.000
Pulmonary involvement	669 (96.0%)	3465 (93.1%)	4134 (93.6%)	1.76 (1.18–2.62)	0.006	1.49 (0.99–2.23)	0.056
Drug-resistant TB	174 (25.0%)	510 (13.7%)	684 (15.5%)	2.09 (1.72–2.55)	0.000	2.13 (1.74–2.59)	0.000
Notification year							
2015	193 (27.7%)	1023 (27.5%)	1216 (27.5%)				
2016	163 (23.4%)	1023 (27.5%)	1186 (26.9%)	0.85 (0.67–1.06)	0.143		
2017	160 (23.0%)	821 (22.1%)	981 (22.2%)	1.03 (0.82–1.30)	0.781		
2018	181 (26.0%)	853 (22.9%)	1034 (23.4%)	1.13 (0.90–1.41)	0.300		

*OR* odds ratio, *CI* confidence interval, *aOR* adjusted odds ratio, *TB* tuberculosis

Among 4417 patients enrolled, 7.2% (316/4417) had Hr-TB, 1.2% (53/4417) had RR-TB, and 4.1% (179/4417) had MDR-TB. The percentages of cases resistant to any fluoroquinolones (FQ) and any second-line injectable drugs (SLID) were 1.7 and 1.0%, respectively. The DR patterns of each age group are shown in Table 3. The percentage of Hr-TB cases among all patients enrolled were 5.4% (40/734), 7.2% (114/1593), and 7.8% (162/2090) in the younger, middle and older age groups, respectively. However, the percentage of Hr-TB in retreatment cases differed significantly among each age group and was highest in the older generation (5.9% (4/68), 5.5% (15/275), 11.3% (40/354), respectively; Table 5). The number of MDR/RR-TB cases (resistant to RIF) was highest in the middle generation, and its proportion decreased significantly as age increased (8.6% (63/734), 6.3% (101/1593), and 3.3% (68/2090), respectively, p = 0.000). This pattern was observed in both new and retreated cases (Tables 4 and 5). Among retreated cases, the percentage of cases resistant to any FQs (ofloxacin, levofloxacin, or moxifloxacin) and any SLIDs (amikacin, kanamycin, or capreomycin) was highest in the younger and middle generations, respectively.

**Table 3:** Drug-resistant profiles of all enrolled patients stratified by age groups

	15–34 (*n* = 734)	35–59 (*n* = 1593)	≥60 (*n* = 2090)	Total (*n* = 4417)	*p-value*
Any drug resistance	118 (16.1%)	258 (16.2%)	308 (14.7%)	684 (15.5%)	0.426
Resistant to					
INH	82 (11.2%)	195 (12.2%)	218 (10.4%)	495 (11.2%)	0.226
RIF	63 (8.6%)	101 (6.3%)	68 (3.3%)	232 (5.3%)	0.000
Rfb	41 (5.6%)	64 (4.0%)	41 (2.0%)	146 (3.3%)	0.000
EMB	22 (3.0%)	65 (4.1%)	44 (2.1%)	131 (3.0%)	0.002
PZA	16 (2.2%)	43 (2.7%)	30 (1.4%)	89 (2.0%)	0.024
Km	4 (0.5%)	14 (0.9%)	13 (0.6%)	31 (0.7%)	0.558
Am	4 (0.5%)	11 (0.7%)	12 (0.6%)	27 (0.6%)	0.876
Cm	5 (0.7%)	12 (0.8%)	17 (0.8%)	34 (0.8%)	0.936
Sm	31 (4.2%)	61 (3.8%)	76 (3.6%)	168 (3.8%)	0.773
Lfx	15 (2.0%)	28 (1.8%)	30 (1.4%)	73 (1.7%)	0.495
Mfx	14 (1.9%)	22 (1.4%)	28 (1.3%)	64 (1.4%)	0.520
Ofx	15 (2.0%)	27 (1.7%)	29 (1.4%)	71 (1.6%)	0.450
Pto	8 (1.1%)	14 (0.9%)	23 (1.1%)	45 (1.0%)	0.785
Cs	1 (0.1%)	3 (0.2%)	2 (0.1%)	6 (0.1%)	0.751
PAS	5 (0.7%)	24 (1.5%)	29 (1.4%)	58 (1.3%)	0.245
INH-monoresistance	40 (5.4%)	114 (7.2%)	162 (7.8%)	316 (7.2%)	0.115
RIF-monoresistance	21 (2.9%)	20 (1.3%)	12 (0.6%)	53 (1.2%)	0.000
Multidrug resistance	42 (5.7%)	81 (5.1%)	56 (2.7%)	179 (4.1%)	0.000
Resistance to any FQs^a^	16 (2.2%)	29 (1.8%)	30 (1.4%)	75 (1.7%)	0.363
Resistance to any SLIDs^b^	7 (1.0%)	16 (1.0%)	19 (0.9%)	42 (1.0%)	0.957

*Abbreviations*: *INH* isoniazid, *RIF* rifampicin, *Rfb* rifabutin, *EMB* ethambutol, *PZA* pyrazinamide, *Km* kanamycin, *Am* amikacin, *Cm* capreomycin, *Sm* streptomycin, *Lfx* levofloxacin, *Mfx* moxifloxacin, *Ofx* ofloxacin, *Pto* prothionamide, *Cs* cycloserine, PAS para-aminosalicylic acid, *FQs* fluoroquinolones, *SLIDs* second-line injectable drugs

^a^Any fluoroquinolones refer to levofloxacin, moxifloxacin, or ofloxacin

^b^Any second-line injectable drugs refer to kanamycin, amikacin, or capreomycin

**Table 4:** Drug-resistant profiles of new patients stratified by age groups

	15–34 (*n* = 666)	35–59 (*n* = 1318)	≥60 (*n* = 1736)	Total (*n* = 3720)	*p-value*
Any drug resistance	91 (13.7%)	189 (14.3%)	230 (13.2%)	510 (13.7%)	0.685
Resistant to					
INH	62 (9.3%)	140 (10.6%)	153 (8.8%)	355 (9.5%)	0.236
RIF	41 (6.2%)	54 (4.1%)	42 (2.4%)	137 (3.7%)	0.000
Rfb	31 (4.7%)	33 (2.5%)	28 (1.6%)	92 (2.5%)	0.000
EMB	15 (2.3%)	38 (2.9%)	29 (1.7%)	82 (2.2%)	0.077
PZA	11 (1.7%)	22 (1.7%)	21 (1.2%)	54 (1.5%)	0.514
Km	2 (0.3%)	5 (0.4%)	11 (0.6%)	18 (0.5%)	0.455
Am	2 (0.3%)	5 (0.4%)	10 (0.6%)	17 (0.5%)	0.584
Cm	3 (0.5%)	5 (0.4%)	15 (0.9%)	23 (0.6%)	0.198
Sm	26 (3.9%)	46 (3.5%)	59 (3.4%)	131 (3.5%)	0.832
Lfx	8 (1.2%)	18 (1.4%)	18 (1.0%)	44 (1.2%)	0.706
Mfx	7 (1.1%)	14 (1.1%)	17 (1.0%)	38 (1.0%)	0.971
Ofx	8 (1.2%)	18 (1.4%)	17 (1.0%)	43 (1.2%)	0.608
Pto	6 (0.9%)	6 (0.5%)	18 (1.0%)	30 (0.8%)	0.196
Cs	0 (0.0%)	1 (0.1%)	1 (0.1%)	2 (0.1%)	0.785
PAS	3 (0.5%)	14 (1.1%)	24 (1.4%)	41 (1.1%)	0.145
INH-monoresistance	36 (5.4%)	99 (7.5%)	122 (7.0%)	257 (6.9%)	0.210
RIF-monoresistance	15 (2.3%)	13 (1.0%)	11 (0.6%)	39 (1.0%)	0.002
Multidrug resistance	26 (3.9%)	41 (3.1%)	31 (1.8%)	98 (2.6%)	0.006
Resistance to any FQs^a^	9 (1.4%)	18 (1.4%)	18 (1.0%)	45 (1.2%)	0.666
Resistance to any SLIDs^b^	5 (0.8%)	6 (0.5%)	17 (1.0%)	28 (0.8%)	0.252

*Abbreviations*: *INH* isoniazid, *RIF* rifampicin, *Rfb* rifabutin, *EMB* ethambutol, *PZA* pyrazinamide, *Km* kanamycin, *Am* amikacin, *Cm* capreomycin, *Sm* streptomycin, *Lfx* levofloxacin, *Mfx* moxifloxacin, *Ofx* ofloxacin, *Pto* prothionamide, *Cs* cycloserine, PAS para-aminosalicylic acid, *FQs* fluoroquinolones, *SLIDs* second-line injectable drugs

^a^Any fluoroquinolones refer to levofloxacin, moxifloxacin, or ofloxacin

^b^Any second-line injectable drugs refer to kanamycin, amikacin, or capreomycin

**Table 5:** Drug-resistant profiles of retreatment patients stratified by age groups

	15–34 (*n* = 68)	35–59 (*n* = 275)	≥60 (*n* = 354)	Total (*n* = 697)	*p-value*
Any drug resistance	27 (39.7%)	69 (25.1%)	78 (22.0%)	174 (25.0%)	0.009
Resistant to					
INH	20 (29.4%)	55 (20.0%)	65 (18.4%)	140 (20.1%)	0.114
RIF	22 (32.4%)	47 (17.1%)	26 (7.3%)	95 (13.6%)	0.000
Rfb	10 (14.7%)	31 (11.3%)	13 (3.7%)	54 (7.7%)	0.000
EMB	7 (10.3%)	27 (9.8%)	15 (4.2%)	49 (7.0%)	0.014
PZA	5 (7.4%)	21 (7.6%)	9 (2.5%)	35 (5.0%)	0.010
Km	2 (2.9%)	9 (3.3%)	2 (0.6%)	13 (1.9%)	0.036
Am	2 (2.9%)	6 (2.2%)	2 (0.6%)	10 (1.4%)	0.131
Cm	2 (2.9%)	7 (2.5%)	2 (0.6%)	11 (1.6%)	0.090
Sm	5 (7.4%)	15 (5.5%)	17 (4.8%)	37 (5.3%)	0.685
Lfx	7 (10.3%)	10 (3.6%)	12 (3.4%)	29 (4.2%)	0.028
Mfx	7 (10.3%)	8 (2.9%)	11 (3.1%)	26 (3.7%)	0.011
Ofx	7 (10.3%)	9 (3.3%)	12 (3.4%)	28 (4.0%)	0.021
Pto	2 (2.9%)	8 (2.9%)	5 (1.4%)	15 (2.2%)	0.393
Cs	1 (1.5%)	2 (0.7%)	1 (0.3%)	4 (0.6%)	0.450
PAS	2 (2.9%)	10 (3.6%)	5 (1.4%)	17 (2.4%)	0.192
INH-monoresistance	4 (5.9%)	15 (5.5%)	40 (11.3%)	59 (8.5%)	0.024
RIF-monoresistance	6 (8.8%)	7 (2.5%)	1 (0.3%)	14 (2.0%)	0.000
Multidrug resistance	16 (23.5%)	40 (14.5%)	25 (7.1%)	81 (11.6%)	0.000
Resistance to any FQs^a^	7 (10.3%)	11 (4.0%)	12 (3.4%)	30 (4.3%)	0.035
Resistance to any SLIDs^b^	2 (2.9%)	10 (3.6%)	2 (0.6%)	14 (2.0%)	0.021

*Abbreviations*: *INH* isoniazid, *RIF* rifampicin, *Rfb* rifabutin, *EMB* ethambutol, *PZA* pyrazinamide, *Km* kanamycin, *Am* amikacin, *Cm* capreomycin, *Sm* streptomycin, *Lfx* levofloxacin, *Mfx* moxifloxacin, *Ofx* ofloxacin, *Pto* prothionamide, *Cs* cycloserine, PAS para-aminosalicylic acid, *FQs* fluoroquinolones, *SLIDs* second-line injectable drugs

^a^Any fluoroquinolones refer to levofloxacin, moxifloxacin, or ofloxacin

^b^Any second-line injectable drugs refer to kanamycin, amikacin, or capreomycin

We further analyzed patterns of resistance to FQs and SLIDs among Hr-TB, RR-TB, and MDR-TB cases. With the exception of first-line anti-TB drugs, resistance to FQs was the highest among that to second-line drugs (Table 6). The percentage of cases resistant to any FQs in Hr-TB, RR-TB, and MDR-TB cases was 1.3% (4/316), 1.9% (1/53), and 20.1% (36/179), respectively. In both non-MDR- and MDR-TB, the percentage of cases resistant to FQs did not differ significantly between age groups (Table 7). Of the 75 cases that were resistant to any FQs, 80.0% (60/75) were resistant to ofloxacin, levofloxacin, and moxifloxacin (Table 8). The percentage of cases resistant to any SLIDs in Hr-TB, RR-TB, and MDR-TB cases was 0.9% (3/316), 1.9% (1/53), and 14.5% (26/179), respectively (Table 6). In both non-MDR- and MDR-TB, the percentage of cases resistant to SLIDs did not differ significantly among age groups (Table 9). Of the 42 cases that were resistant to any SLIDs, 64.3% (27/42) were resistant to both kanamycin and amikacin (Table 10).

**Table 6:** Drug-resistant profiles of 684 drug-resistant tuberculosis cases stratified by resistance to isoniazid and rifampicin

	Other resistant TB (*n* = 136)	Hr-TB (*n* = 316)	RR-TB (*n* = 53)	MDR-TB (*n* = 179)	Total (*n* = 684)
Resistant to					
INH	0 (0.0%)	316 (100.0%)	0 (0.0%)	179 (100.0%)	495 (72.4%)
RIF	0 (0.0%)	0 (0.0%)	53 (100.0%)	179 (100.0%)	232 (33.9%)
Rfb	0 (0.0%)	0 (0.0%)	36 (67.9%)	110 (61.5%)	146 (21.3%)
EMB	9 (6.6%)	27 (8.5%)	1 (1.9%)	94 (52.5%)	131 (19.2%)
PZA	16 (11.8%)	6 (1.9%)	1 (1.9%)	66 (36.9%)	89 (13.0%)
Km	4 (2.9%)	2 (0.6%)	1 (1.9%)	24 (13.4%)	31 (4.5%)
Am	3 (2.2%)	2 (0.6%)	1 (1.9%)	21 (11.7%)	27 (3.9%)
Cm	11 (8.1%)	2 (0.6%)	1 (1.9%)	20 (11.2%)	34 (5.0%)
Sm	64 (47.1%)	43 (13.6%)	2 (3.8%)	59 (33.0%)	168 (24.6%)
Lfx	34 (25.0%)	4 (1.3%)	1 (1.9%)	34 (19.0%)	73 (10.7%)
Mfx	29 (21.3%)	3 (0.9%)	1 (1.9%)	31 (17.3%)	64 (9.4%)
Ofx	32 (23.5%)	4 (1.3%)	1 (1.9%)	34 (19.0%)	71 (10.4%)
Pto	0 (0.0%)	25 (7.9%)	0 (0.0%)	20 (11.2%)	45 (6.6%)
Cs	0 (0.0%)	1 (0.3%)	0 (0.0%)	5 (2.8%)	6 (0.9%)
PAS	2 (1.5%)	29 (9.2%)	0 (0.0%)	27 (15.1%)	58 (8.5%)
Resistance to any FQs^a^	34 (25.0%)	4 (1.3%)	1 (1.9%)	36 (20.1%)	75 (11.0%)
Resistance to any SLIDs^b^	12 (8.8%)	3 (0.9%)	1 (1.9%)	26 (14.5%)	42 (6.1%)

*Abbreviations*: *INH* isoniazid, *RIF* rifampicin, *Rfb* rifabutin, *EMB* ethambutol, *PZA* pyrazinamide, *Km* kanamycin, *Am* amikacin, *Cm* capreomycin, *Sm* streptomycin, *Lfx* levofloxacin, *Mfx* moxifloxacin, *Ofx* ofloxacin, *Pto* prothionamide, *Cs* cycloserine, *PAS* para-aminosalicylic acid, *FQs* fluoroquinolones, *SLIDs* second-line injectable drugs

^a^Any fluoroquinolones refer to levofloxacin, moxifloxacin, or ofloxacin

^b^Any second-line injectable drugs refer to kanamycin, amikacin, or capreomycin

**Table 7:** Proportions of fluoroquinolone resistance stratified by age groups

	15–34	35–59	≥60	Total	*p-value*
Non-multidrug-resistance TB	692 (100.0%)	1512 (100.0%)	2034 (100.0%)	4238 (100.0%)	
Resistance to any FQ^a^	5 (0.7%)	15 (1.0%)	19 (0.9%)	39 (0.9%)	0.824
Ofx resistance	4 (0.6%)	15 (1.0%)	18 (0.9%)	37 (0.9%)	0.623
Lfx resistance	5 (0.7%)	15 (1.0%)	19 (0.9%)	39 (0.9%)	0.824
Mfx resistance	4 (0.6%)	12 (0.8%)	17 (0.8%)	33 (0.8%)	0.798
Multidrug-resistance TB	42 (100.0%)	81 (100.0%)	56 (100.0%)	179 (100.0%)	
Resistance to any FQ^a^	11 (26.2%)	14 (17.3%)	11 (19.6%)	36 (20.1%)	0.502
Ofx resistance	11 (26.2%)	12 (14.8%)	11 (19.6%)	34 (19.0%)	0.309
Lfx resistance	10 (23.8%)	13 (16.0%)	11 (19.6%)	34 (19.0%)	0.576
Mfx resistance	10 (23.8%)	10 (12.3%)	11 (19.6%)	31 (17.3%)	0.241

*Abbreviations*: *TB* tuberculosis, *FQ* fluoroquinolone, *Ofx* ofloxacin, *Lfx* levofloxacin, *Mfx* moxifloxacin

^a^Any fluoroquinolones refer to levofloxacin, moxifloxacin, or ofloxacin

**Table 8:** Drug-resistant profiles of different fluoroquinolones stratified by multidrug-resistant status

	Ofx	Lfx	Mfx	n (%)
Non-multidrug-resistance TB (*n* = 39)				
	R	R	R	31 (79.5%)
	R	R	S	6 (15.4%)
	S	R	R	2 (5.1%)
Multidrug-resistance TB (*n* = 36)				
	R	R	R	29 (80.6%)
	R	R	S	3 (8.3%)
	R	S	S	2 (5.6%)
	S	R	R	2 (5.6%)

*Abbreviations*: *TB* tuberculosis, *Ofx* ofloxacin, *Lfx* levofloxacin, *Mfx* moxifloxacin, *R* resistant, *S* sensitive

**Table 9:** Proportions of resistance to second-line injectable drugs and streptomycin stratified by age groups

	15–34	35–59	≥60	Total	*p-value*
Non-multidrug-resistance TB	692 (100.0%)	1512 (100.0%)	2034 (100.0%)	4238 (100.0%)	
Resistance to any SLID^a^	2 (0.3%)	2 (0.1%)	12 (0.6%)	16 (0.4%)	0.082
Km resistance	0 (0.0%)	1 (0.1%)	6 (0.3%)	7 (0.2%)	0.127
Am resistance	0 (0.0%)	1 (0.1%)	5 (0.2%)	6 (0.1%)	0.207
Cm resistance	2 (0.3%)	2 (0.1%)	10 (0.5%)	14 (0.3%)	0.179
Sm resistance	16 (2.3%)	37 (2.4%)	56 (2.8%)	109 (2.6%)	0.761
Multidrug-resistance TB	42 (100.0%)	81 (100.0%)	56 (100.0%)	179 (100.0%)	
Resistance to any SLID^a^	5 (11.9%)	14 (17.3%)	7 (12.5%)	26 (14.5%)	0.633
Km resistance	4 (9.5%)	13 (16.0%)	7 (12.5%)	24 (13.4%)	0.585
Am resistance	4 (9.5%)	10 (12.3%)	7 (12.5%)	21 (11.7%)	0.878
Cm resistance	3 (7.1%)	10 (12.3%)	7 (12.5%)	20 (11.2%)	0.638
Sm resistance	15 (35.7%)	24 (29.6%)	20 (35.7%)	59 (33.0%)	0.690

*Abbreviations*: *TB* tuberculosis, *SLID* second-line injectable drug, *Km* kanamycin, *Am* amikacin, *Cm* capreomycin, *Sm* streptomycin

^a^Any second-line injectable drugs refer to kanamycin, amikacin, or capreomycin

**Table 10:** Drug-resistant profiles of different second-line injectable drugs stratified by multidrug-resistant status

	Cm	Km	Am	n (%)
Non-multidrug-resistance TB (*n* = 16)				
	R	R	R	5 (31.3%)
	R	S	S	9 (56.3%)
	S	R	R	1 (6.3%)
	S	R	S	1 (6.3%)
Multidrug-resistance TB (*n* = 26)				
	R	R	R	17 (65.4%)
	R	R	S	1 (3.8%)
	R	S	S	2 (7.7%)
	S	R	R	4 (15.4%)
	S	R	S	2 (7.7%)

*Abbreviations*: *TB* tuberculosis, *Km* kanamycin, *Am* amikacin, *Cm* capreomycin, *R* resistant, *S* sensitive

## Discussion

This is the first study to compare percentages of DR-TB cases among various age groups in South Korea. The percentage of MDR/RR-TB cases was the highest in the younger generation. The percentage of Hr-TB cases did not differ among the various age groups; however, it was higher among retreated patients in the older generation than that in the younger generation. Among the second-line anti-TB drugs, the percentage of cases resistant to any FQs was the highest and was similar among the various age groups. DR-TB is characterized by unfavorable outcome such as treatment failure, loss to follow-up and death, and leads to the spread of drug resistant organisms in the community as a result of inefficient interactions between the National Tuberculosis Control Program and patients with TB [4]. Therefore, understanding the prevalence and trends of drug resistance may help to identify TB treatment failures and determine the direction of future TB treatment policies.

MDR/RR-TB is a global public health concern and an important target for national TB control programs in many countries, including South Korea. According to a recent WHO report [13], an estimated 3.4% of new and 18% of previously treated TB cases were MDR/RR-TB. In South Korea, a recent study [5] revealed that the percentage of MDR/RR-TB among new and retreated cases between 2010 and 2014 decreased from 6.3 to 3.5% and from 29.4 to 19.2%, respectively. Similarly, in our study, 3.7% (137/3720) and 13.6% (95/697) of new and retreated cases, respectively, were MDR/RR-TB. Although INH is an important drug which is safe and affordable, problems with Hr-TB have been neglected by the TB community [14]. The global percentage of Hr-TB cases is 7.2% of new and 11.6% of previously treated TB cases [13]. In our study, the number of INH-resistant cases was higher than that of RIF-resistant cases among all TB patients, with 6.9% (257/3720) and 8.5% (59/697) of new and retreated cases, respectively, being Hr-TB. The previous nationwide survey of anti-TB DR conducted in 2004 showed that the prevalence of Hr-TB among new and retreated cases was 5.1 and 6.8%, respectively, in South Korea [4]. Hr-TB and MDR/RR-TB cases should be continuously surveilled in order to assess their prevalence.

The great economic development and rapid decline of TB incidence observed during the last 50 years may have various impacts on drug resistance profiles in various age group. Korean patients born before 1950 likely experienced the explosion of the TB epidemic after the Korean War. During economic growth between the 1960s and 1970s, TB prevalence declined with the implementation of the national TB control program in 1960 [6]. In the 1960s and 1970s, triple therapy including INH and streptomycin was administered for 18 months. Indiscriminate use of anti-TB drugs and lack of patient management between the 1950s and 1970s may have led to the emergence of resistance to INH and streptomycin. Accordingly, our study revealed that among retreated cases, the percentage of Hr-TB cases in the older generation was the highest and almost twice that of the percentage in the younger and middle generations. This may be related to past exposure, including long-term and improper use of INH. Furthermore, INH resistance formed the highest percentage of all drug resistant cases in both new and retreated patients.

Since the 1980s, TB prevalence in South Korea decreased significantly compared to that in previous decades. In addition to having access to sustained economic development and universal health coverage, patients born after 1980 had a low chance of becoming infected with TB. A shorter regimen that included RIF and was administered for 6 to 9 months was also introduced in the 1980s. Because of no exposure of a RIF-resistant strain before the 1980s, reactivation from remote RR-TB infection would be less likely in the older generations. We can assume that currently observed RIF-resistant cases in South Korea are a mixture of reactivation from remote TB infections with strains in the early years of RIF introduction and recent infection. Our study showed that younger patients showed a higher percentage of RIF-resistant cases. This trend was consistent in both new and retreated patient groups, suggesting high rates of primary infection with MDR/RR-TB and acquired RIF resistance among young Korean patients. Although it is generally thought that a large percentage of MDR-TB cases arise from de novo resistance selection during previous TB treatment, the predominant incident MDR-TB etiology has now shifted to direct person-to-person MDR strain transmission [15]. A recent study suggested that > 80% of incident MDR-TB cases in most present-day epidemic settings result from transmission of MDR-TB [16]. Therefore, to control the MDR-TB epidemic in young patients, primary MDR-TB transmission and infection control and appropriate patient management should be prioritized in South Korea.

According to the revised WHO DR-TB treatment guidelines [17], levofloxacin is essential to MDR/RR- and Hr-TB treatment. FQs are widely used antimicrobial agents in out- and in-patient treatment, and its use in patients with TB at a single tertiary hospital in South Korea, regardless of their DR status, was also high [18]. Here, the percentage of FQ-resistant cases was the highest among that to second-line drugs, especially in young patients with prior anti-TB treatment history. In our study population, proportion of FQ resistance in both RR-TB and Hr-TB was low at 1.9 and 1.3%, respectively, which implies safe addition of levofloxacin to regimens according to the revised WHO guideline [17]. However, 26% of young patients with MDR-TB in our study population showed resistance to any FQs, implying a high public health burden in the younger generations. In addition, 25% of RIF- and INH-susceptible patients were resistant to any FQs, which is higher than results reported in a recent multi-country surveillance study [19]. Such a high prevalence of FQ resistance may be due to the widespread use of FQs in various clinical settings [18]. Several studies showed that FQ exposure prior to TB diagnosis was associated with FQ resistance [20]. Therefore, the implementation of FQ prescription antibiotic stewardship programs for drug-susceptible TB should be considered in South Korea.

This study had several limitations. First, although we hypothesized that drug resistance profiles may differ among various age groups due to rapid and intense socioeconomic changes in late twentieth century in South Korea, an age-period-cohort analysis, the better strategy to identify period and cohort effects on health, needs to be further performed to complement our study [21]. The accumulation of repetitive cross-sectional data regarding drug resistance is necessary to perform such long-term analysis. Second, our results do not represent overall drug resistance in South Korea. In addition, more complex cases, such as drug-resistant TB, might have been notified, because participated hospitals were university-affiliated. However, because these hospitals are broadly located in several administrative districts and record approximately 2200 TB cases annually (almost 5% of all TB cases notified in South Korea), our study may reflect anti-TB drug resistance strains in South Korea. Third, the cause of high RIF resistance prevalence in the younger age group was not identified. Due to its retrospective design, detailed data regarding prior anti-TB treatment and clinical information were not available here. It has been reported that the younger-age group (< 30) clusters more frequently than the older-age group (> 50) based on molecular epidemiological tools [22]. Because of small numbers of RIF-resistant cases enrolled in our study, this unique clustering features among the younger age group might have influenced the high RIF resistance prevalence. Further epidemiological investigations including molecular and genomic typing may elucidate TB transmission routes and identify possible strategies. Lastly, causes of DR-TB are multifactorial, such as undernutrition.

## Conclusions

We showed that anti-TB drug resistance profiles differ among patients in various age groups, with a high proportion of RIF and INH resistance in the young and elderly patient groups, respectively. Among young patients, emerging FQ resistance and MDR/RR-TB may limit anti-TB treatment strategies, and this should be regarded as a warning against the widespread use of FQ in the community [5]. In establishing future TB policies and treatment guidelines, differences in drug resistance patterns among age groups should be considered.

## Data Availability

All data generated or analyzed during this study are included in this published article.

## Abbreviations

CI: Confidence interval
DR-TB: Drug-resistant tuberculosis
DST: Drug-susceptibility test
FQ: fluoroquinolone
Hr-TB: Isoniazid-mono-resistant TB
INH: Isoniazid
MDR-TB: Multidrug-resistant TB
OR: odds ratio
RIF: Rifampicin
RR-TB: Rifampicin-mono-resistant TB
SLID: Second-line injectable drug
TB: Tuberculosis
